# Identification of Two Subsets of Murine DC1 Dendritic Cells That Differ by Surface Phenotype, Gene Expression, and Function

**DOI:** 10.3389/fimmu.2021.746469

**Published:** 2021-10-26

**Authors:** David Hongo, Pingping Zheng, Suparna Dutt, Rahul D. Pawar, Everett Meyer, Edgar G. Engleman, Samuel Strober

**Affiliations:** ^1^ Department of Medicine, Division of Immunology and Rheumatology, Stanford University School of Medicine, Stanford, CA, United States; ^2^ Department of Medicine, Division of Blood and Marrow Transplantation, Stanford University School of Medicine, Stanford, CA, United States; ^3^ Department of Pathology, Stanford University School of Medicine, Stanford, CA, United States

**Keywords:** dendritic cells, type I dendritic cells, type II dendritic cell, CD4 T cell, CD8 T cell, tumor vaccination

## Abstract

Classical dendritic cells (cDCs) in mice have been divided into 2 major subsets based on the expression of nuclear transcription factors: a CD8^+^Irf8^+^Batf3 dependent (DC1) subset, and a CD8^-^Irf4^+^ (DC2) subset. We found that the CD8^+^DC1 subset can be further divided into CD8^+^DC1a and CD8^+^DC1b subsets by differences in surface receptors, gene expression, and function. Whereas all 3 DC subsets can act alone to induce potent Th1 cytokine responses to class I and II MHC restricted peptides derived from ovalbumin (OVA) by OT-I and OT-II transgenic T cells, only the DC1b subset could effectively present glycolipid antigens to natural killer T (NKT) cells. Vaccination with OVA protein pulsed DC1b and DC2 cells were more effective in reducing the growth of the B16-OVA melanoma as compared to pulsed DC1a cells in wild type mice. In conclusion, the Batf3-/- dependent DC1 cells can be further divided into two subsets with different immune functional profiles *in vitro* and *in vivo*.

## Highlights

Two subsets of DC1 dendritic cells differ by surface phenotype, gene expression, and function.

## Introduction

The subset of CD8^+^dendritic cells (DCs) that is dependent on the Batf3 and Irf8 nuclear transcription factors for development and maturation in mouse lymphoid tissues has been extensively studied (1–5). This subset, that has been identified as DC1 by Murphy and co-workers (6, 7) plays a required role in the induction of CD8^+^ T cell immunity to tumors and viruses (8–15). DC1 cells express the surface markers CD11c and MHCII associated with all classical myeloid DCs, and express high levels of CD24 and XCR1 and low levels of CD172 (SIRPα) surface markers (5–16). This surface phenotype has been used to distinguish the DC1 subset from the CD8^-^ DC2 subset that expresses low levels of CD24 and XCR1 and high levels of CD172a (6, 7). Inactivation of the gene encoding Batf-3 results in the selective elimination of CD8^+^ and CD103^+^ DC1 DCs (17, 18). Both subsets express high levels of XCR1, low levels of CD172, and can stimulate CD8+ T cell immunity (19–21). In contrast to the DC1 subset, the DC2 subset is dependent on the Irf4 nuclear transcription factor and has been subdivided further into Notch2-dependent and KIf4-dependent populations (22, 23). Batf3 is expressed in both the Irf8^+^ and Irf4^+^ DCs (5). Nevertheless, the DC2 cells are predominantly Batf3 independent (20).

It is not clear whether the CD8^+^ Batf-3 dependent DC1 subset is homogeneous or whether it can be divided further into additional subsets. Phenotypic and functional heterogeneity of the subset has been described previously with regard to the expression of CD103 in the spleen, skin draining lymph nodes, tumors, and intestines (24–28). DC1 cells in the skin draining lymph nodes, tumors, and intestines express high levels of CD103 whereas DC1 cells in the spleen have low or undetectable levels (24–28). The CD8^+^ subset of DCs has been reported to be more potent than the CD8^-^ subset in cross-presenting cell associated protein antigens to conventional CD8^+^ T cells, and selective depletion of the CD8+ subset markedly attenuates CD8+ T cell immunity to tumors and viruses by virtue of cross antigen presentation (29–32). CD8^+^ DCs take up proteins from exogenous cell sources that are the source of the antigens, such as tumor cells and viral infected cells, process the proteins such that the derived peptides are cross-presented to CD8^+^ T cells after association with MHC receptors on the cell surface (1–11). In accordance with these observations, the ability of radiation therapy to induce T cell mediated durable complete remissions of solid and lymphoid tumors was dependent on the presence of CD8^+^Batf3 dependent DCs, since remissions observed in wild type mice were abrogated in *Batf3* ^-/-^ mice (33, 34). Remissions were restored by injecting CD8^+^ DCs into the tumors (33, 34).

Previous studies showed that after uptake of the soluble ovalbumin protein injected *in vivo*, the CD8^+^ DCs were effective at presenting the antigen to ovalbumin specific TCR transgenic CD8^+^T cells but not CD4^+^ T cells, and CD8^-^DCs were effective at presenting to transgenic CD4^+^T cell (32). These observations are consistent with the dominant role of CD8^+^DCs in the induction of CD8^+^T cell immunity to cell bound viral and tumor antigens. However, vaccination with soluble ovalbumin protein linked to antibodies directed to the DCIR2 surface receptor of the CD8^-^ subset of DCs induced effective CD8^+^T cell immunity against the B16-OVA melanoma tumor after injection of the conjugate *in vivo* (35). Thus, CD8^-^DC2 cells can also induce CD8^+^T cell immunity to an ovalbumin tumor antigen depending on the nature of the vaccination. However, it was not determined whether CD8^+^DCs in the tumor microenvironment were also required for the CD8^+^ T cell anti-tumor effect in addition to the systemic immune response induced by the DCIR2 targeted CD8^-^ DCs.

The CD8^+^DC1 and CD8^-^DC2 subsets have also been compared for their efficacy in presenting glycolipid antigens to NKT cells (36, 37). The latter T cells recognize glycolipids in association with the CD1d antigen presenting molecule (38–40). The DC1 subset was effective and the DC2 subset was ineffective in the induction of NKT cell anti-glycolipid immune responses. It is not clear whether the ability of the CD8^+^ DC1 subset to present soluble and cell associated protein antigens to CD8^+^ T cells, on one hand, and glycolipid antigens to NKT cells, on the other, is a function of a single subset or of at least two subsets of CD8^+^ DC1 cells.

The current study identified two major subsets of Batf3 dependent CD8^+^ DCs (DC1a and DC1b) that differ from each other and from the CD8^-^DC2 subset on the basis of clear differences in their surface receptors, and gene expression. The ability of all of the latter subsets to induce T cell immune responses to a variety of antigens was compared, and key differences were elucidated.

## Results

### Identification of 2 Subsets of CD8^+^ Batf3 Dependent DCs

**Figure 1:**
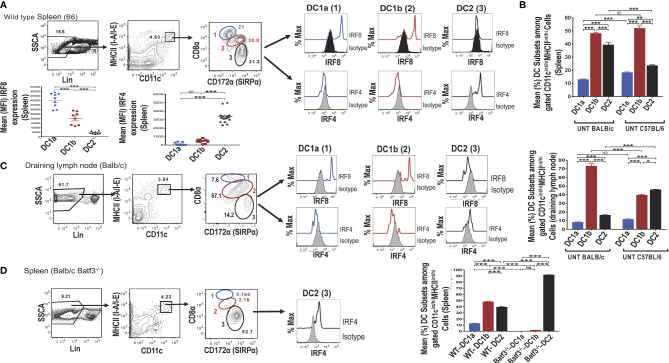
Identification of 2 subsets of CD8^+^ Batf3 dependent DCs. Spleen cells from untreated wild-type (WT) C57BL/6 or BALB/c mice were stained and analyzed by flow cytometry for DC susbsets. **(A)** Show representative two color FACS gating strategy to identify MHCII(I-A/I-E)^+^ CD11+ total DCs after gating on lineage negative (CD3, B220, CD19, CD56, Gr-1) spleen cells. Boxes in panels show the percentages of cells enclosed. Gated total DCs cells were further analyzed for CD8 *versus* CD172a, and 3 discrete populations were observed; populations 1 (DC1a), 2 (DC1b), and 3 (DC2) (indicated by blue, red, and black ovals) respectively. The 3 populations were analyzed for intracellular staining of the Irf8 and Irf4 nuclear factors compared to isotype control staining. Representative histograms are shown as well as the mean (MFI) for Irf8 and Irf4 staining intensity among the gated DC populations (n = 8-10). One-way ANOVA (Holm-Sidak’s multiple comparison test) was used for statistical analysis. *p < 0.05, **p < 0.01, ***p < 0.001, NS-not significant (p > 0.05). **(B)** Compares the mean (± SEM) percentages of populations 1 (DC1a), 2 (DC1b), and 3 (DC2) among total DCs from the spleens of untreated (UNT) WT C57BL/6 and BALB/c mice (n =18-20). **(C)** Shows staining for CD8α *versus* CD172 on gated CD11c^+^MHCII^+^ draining lymph node cells from WT BALB/c mice. The 3 gated populations of DCs were stained for Irf8 and Irf4, and representative histograms are shown. The bar graphs compare the mean percentages of DC1a, DC1b, and DC2 cells from draining lymph nodes of WT BALB/c and C57BL/6 mice **(D)** Shows representative two color FACS patterns of CD11c+ MHCII+ cells among spleen cells from *Batf3* ^-/-^ BALB/c mice. Gated cells from each box were further analyzed for CD8 *versus* CD172a. Too few populations 1 and 2 cells were available to stain for nuclear factors, and expression of Irf4 by population 3 is shown in a representative histogram. The mean percentages of the 3 DC populations are compared from WT *versus* Batf3-/- BALB/c mice in the bar graphs.

Single cells were harvested from the spleen of adult wild type (WT) C57BL/6 mice, stained for surface receptors, and CD3-CD56-CD3-CD19-Gr-1- (Lin-) cells were analyzed by flow cytometry for CD11c vs MHC class II expression. As shown in (**Figure 1A**), 4.5% were contained in the box enclosing a discrete population of CD11c^+^MHCII^+^ cells that were identified as dendritic cells (DCs). Further study of the gated DCs in the spleen of WT mice for expression of CD8 vs CD172a showed that 3 populations of cells with discrete concentric contours were present (enclosed in ellipses in **Figure 1A**). Population 1 (identified as the DC1a subset in all subsequent Figures and Tables) contained CD8^hi^CD172^lo^ cells, population 2 (identified as the DC1b subset) contained CD8^int^CD172^int^ cells, and population 3 (identified as DC2 subset) contained CD8^-^CD172^hi^ cells. In order to determine whether populations 1 and 2 expressed the Irf8+/Irf4- nuclear factor expression pattern that characterizes DC1cells, all three populations were gated separately, and stained for intracellular Irf8 and Irf4. As shown in the histograms in **Figure 1A**, both the DC1a and DC1b populations expressed the Irf8+/Irf4- pattern. In contrast, the DC2 population, expressed the Irf8-Irf4+ pattern that has been shown previously to characterize the DC2 subset. The mean percentages of the 3 populations among gated CD11c^+^MHC^+^ cells enclosed in the ellipses in the spleen of C57BL/6 WT mice are shown in the panel of **Figure 1B**. DC1a, DC1b, and DC2 subsets made up about 12%,48%, and 40% of total DCs respectively. In additional experiments, the mean percentages of the 3 subsets among splenic DCs in C57BL/6 and BALB/c WT mice were compared. The DC1b cells represented the majority of DCs in both strains, and the mean percentage of DC2 cells was significantly higher in the BALB/c mice.

The mean fluorescence intensity (MFI) of staining for Irf8 and Irf4 among the 3 populations of gated splenic DCs is shown in **Figure 1A**. Mean intensity for Irf8 was highest in the DC1a cells, intermediate in DC1b cells and at background levels in DC2 cells. Differences in means were statistically significant for all 3 populations (p<0.001). As expected, the staining pattern for Irf4 showed the opposite pattern with the mean for Irf4 significantly increased (p<0.001) as compared to background staining levels for DC1a and DC1b cells.

Similar analysis of expression of CD8 vs CD172 on gated DCs from the draining lymph nodes of WT BALB/c mice showed that the same discrete contours for 3 populations was observed, and histograms of Irf8 and Irf4 staining followed the pattern observed in the spleen (**Figure 1C**). Although the mean percentage of DC1b cells in the draining nodes of BALB/c mice was about 70%, the DC2 cells constituted the majority of DCs in the draining nodes of C57BL/6 mice (**Figure 1C**).

Additional analysis of gated DCs from the spleen of Batf3-/- BALB/c mice showed a dramatic reduction of the DC1a and DC1b cells in representative histograms, and the DC2 cells with the Irf8-Irf4+ pattern accounted for about 93% of total DCs (**Figure 1D** **)**. The DC1a and DC1b population each made up means of less than 3% of the gated DCs, and the DC2 cells made up a mean of over 90% (of **Figure 1D**). Mean percentages of each subset are compared for BALB/c WT *versus* Batf3-/- mice in the bar graph in **Figure 1D**. Mean percentages for DC1a and DC1b cells in the latter mice were below 2% and the mean for DC2 cells was above 90%. The loss of DC1a and DC1b subsets indicated that these were CD8^+^Batf3 dependent DCs that have been previously identified as DC1 cells (6, 7). The DC2 subset was similar to the CD8^-^CD172^hi^ subset previously identified as the DC2 subset (6, 7).

### DC1a, DC1b and DC2 Cells Differ in Their Expression of Multiple Surface Markers

**Figure 2:**
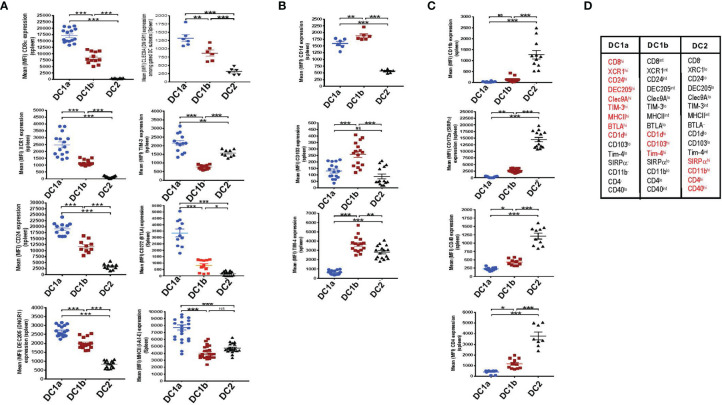
DC1a and DC1b cells in the spleen differ in their expression of multiple surface markers that also distinguish them from DC2 cells. **(A)** Shows individual and mean (MFI) levels of staining intensity of receptors that are most highly expressed on gated population 1(*DC1a*) cells including CD8a, XCR1, CD24, DEC205, Clec9A, TIM-3, MHCII, and BTLA (CD272) in WT BALB/c spleen cell samples. **(B)** Shows MFI levels of receptors most highly expressed on population 2 (*DC1b*) cells including CD1d, CD103, (DNGR1), and TIM-4. **(C)** Shows MFI levels of receptors most highly expressed on population 3 (*DC2*) cells including CD11b, CD172a (SIRP), CD40, and CD4. **(D)** Table shows the summary patterns of high expression of surface receptors on 3 DC subsets in the spleen. Receptors in red are those with MFIs that were significantly increased as compared to that of the two other DC populations. (N = 10-20). One-way ANOVA (Holm-Sidak’s multiple comparison test) was used for statistical analysis. *p < 0.05, **p < 0.01, ***p < 0.001, NS, not significant (p > 0.05). MFI, mean fluorescence intensity.

In an effort to further distinguish the DC populations from one another, we stained the 3 gated subsets of DCs in the WT BALB/c spleen for a variety of additional surface receptors including those previously shown to identify DC1 cells such as XCR1 and CD24 (5–7). **Figure 2A** shows that XCR1, CD24, DEC205, Tim-3, Clec9A, BTLA, MHCII, and CD8 were highly expressed on DC1a cells as compared to other DC populations (MFI vs DC1b and DC2 cells p<0.001). In contrast, the MFI for CD1d, CD103, and Tim4 on DC1b cells was significantly increased as compared to that of DC1a and DC2 cells (**Figure 2B**). Surface receptors highly expressed on DC2 cells as compared to DC1a and DC1b cells were CD11b, CD40, CD172, and CD4 (p<0.001) (**Figure 2C**). **Figure 2D** summarizes the patterns of high levels of receptor expression that distinguishes each of the 3DC populations.

### DC1a, DC1b and DC2 Cells Differ in Their Tissue Distributions

**Figure 3:**
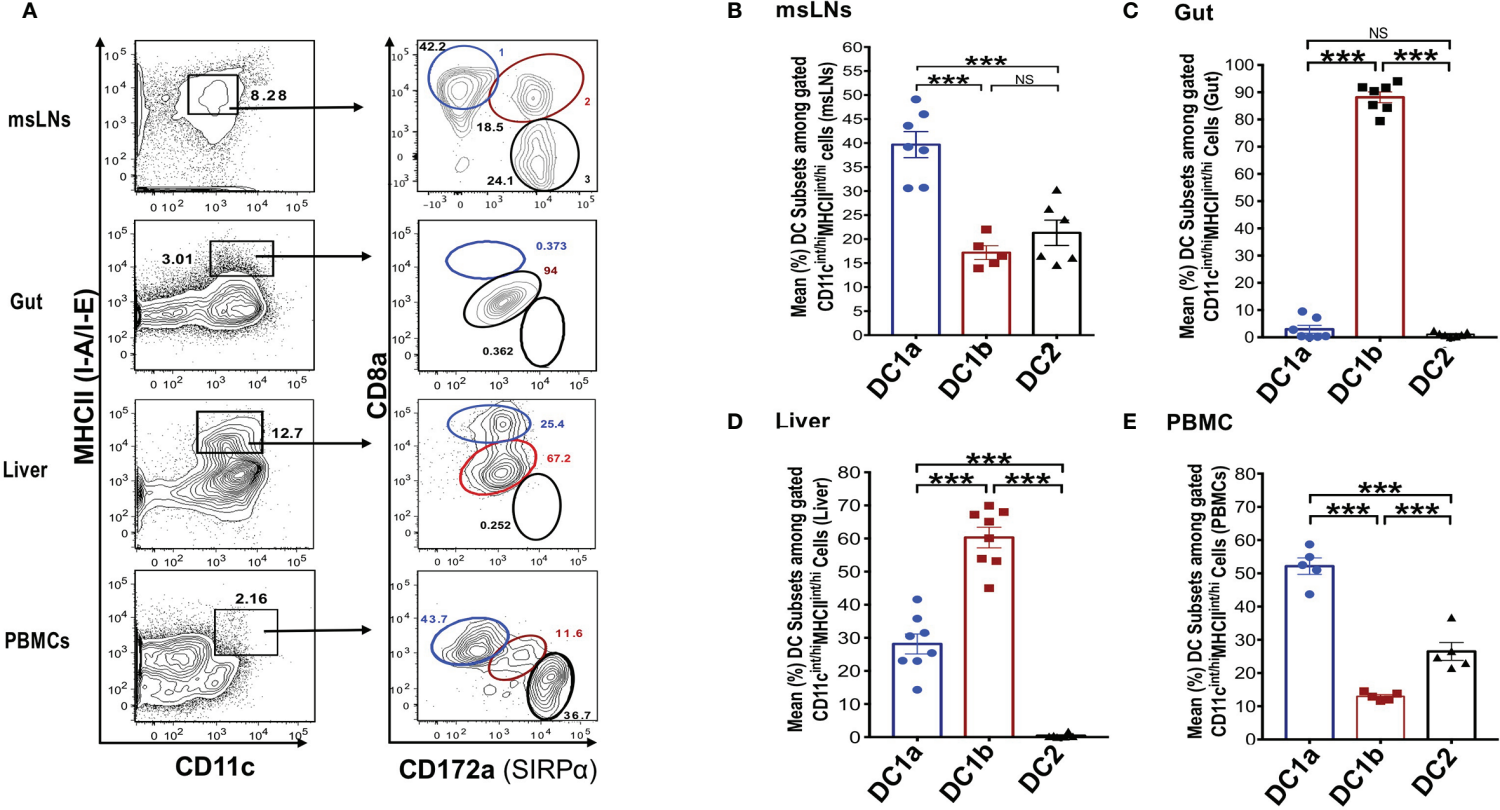
Distribution of 3 DC subsets in different tissues (mesenteric LNs, gut, liver and PBMCs). **(A)** Shows representative FACS staining of gated MHCII+CD11c+ total DCs from mesenteric lymph nodes (msLNs), intestines (gut), liver and PBMCs of WT BALB/c mice. Total DCs were further analyzed for CD172a *versus* CD8a staining, and ellipses outline populations 1,2, and 3(DC1a, DC1b, DC2). Mean (SEM) percentages of 3 DC populations among total DCs in **(B)** msLNs, **(C)** gut, **(D)** liver, and **(E)** PBMCs cells. (N = 6-10). One-way ANOVA (Holm-Sidak’s multiple comparison test) was used for statistical analysis. ***p < 0.001, NS, not significant.

In further studies, we elucidated the distribution of the DC populations in the mesenteric lymph nodes (msLNs), intestines (gut), liver, and peripheral blood. Mononuclear cells were collected from the four tissue sources from WT BALB/c mice, and the percentages of these cells among total DCs were determined and compared to that in the spleen and draining lymph nodes. MHCII+CD11c+ total DCs from each tissue were gated as before and analyzed for the expression of CD8 *versus* CD172 as shown in **Figure 3A**. Three DC populations were clearly identified among the the msLN cells and peripheral blood mononuclear cells (PBMC) as shown in the representative two color stainings. In contrast, only one population of DCs that had the staining characteristics of DC1b cells was found among the gut mononuclear cells, and neither the DC1a nor the DC2 populations were identified (**Figure 3A**). Two DC populations with characteristics of the DC1a and DC1b cells were found among liver mononuclear cells, and the DC2 population was not observed (**Figure 3A**).

**Figures 3B-E** shows the mean percentages of each DC population among total DCs from the 4 tissues. Each population accounted for a mean of at least 10% among total DCs in the msLNs and PBMC, and the mean percentage of DC1a cells were significantly increased as compared to the other populations in these tissues (p<0.001) (**Figures 3B, E**). The mean percentage of DC1a and DC2 cells among total DCs in the gut accounted for less that 2%, and almost all were DC1b cells (**Figure 3C**). The mean percentage of DC2 cells was below 1% in the liver, and about 60% were DC1b cells and 30% DC1a (**Figure 3D**). In summary, three populations of DCs were found in the msLNs and PBMC as was observed in the spleen and draining lymph nodes. Whereas the dominant population in the latter two tissues was DC1b, the dominant population among the msLNs and the PBMC was DC1a. DC2 cells were not identified in the two non-lymphoid tissues (gut and liver), and were easily identified in the four lymphoid tissues.

### Gene Expression Profiling Distinguishes the 3 DC Populations

**Figure 4:**
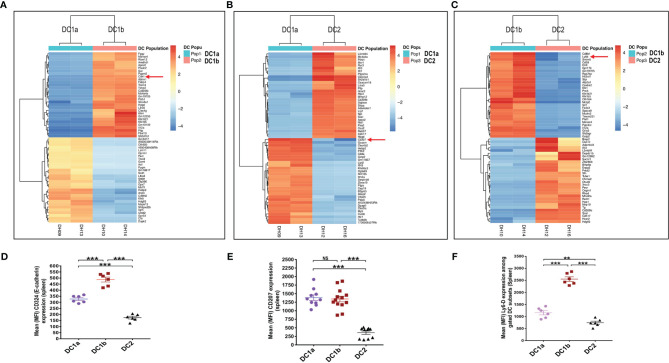
Gene expression profiling reveals additional differences between the 3 DC subsets. **(A)** Heat map of genes with significantly increased or decreased expression levels (as determined by RNA-seq analysis) in sorted DC1a (Pop 1) *versus* DC1b (Pop 2) or **(B)** DC1a (Pop 1) *versus* DC2 (Pop 3) or **(C)** DC1b (Pop 2) *versus* DC2 (Pop 3) from the WT BALB/c spleen. Each row represents a gene identified on the right side of the map, and each column represents a result from a single sample. The color scale shown on the map shows the relative expression levels of the genes across all the samples. Red and yellow shades represent higher expression levels, while blue shades represent lower expression levels. **(D–F)** Representative staining intensities and MFI of upregulated receptors CD207 (langerin), CD324 (E-cadherin; *Cdh1*), and Ly6-D cells on DC subsets (n = 10-20). The genes of these three markers were identified by the RNAseq analysis and were stained to confirm their expression at the protein level. One-way ANOVA (Holm-Sidak’s multiple comparison test) was used for statistical analysis of MFI. *p < 0.05, **p < 0.01, ***p < 0.001, NS, not significant (p > 0.05).

In order to compare the gene expression pattern of the 3 DC populations in the spleen of WT BALB/c mice, the cells were sorted by flow cytometry according to the gates shown in **Figure 1A** for populations 1, 2, and 3, RNA was extracted from the sorted cells, and the gene expression profiles were compared using the RNAseq. **Figure 4A** shows the heat map comparison of gene expression for sorted DC1a vs DC1b cells, **Figure 4B** shows the comparison for DC1a vs DC2 cells, and **Figure 4C** shows the comparison for DC1b vs DC2 cells. The 3 heat maps clearly distinguished the gene expression patterns of the 3 sorted populations. Further quantitative analysis of differences in the immune response and signaling pathways encoded by the upregulated genes of the DC1b *versus* DC1a cells are shown in **Supplemental Figure 1**.

In particular, genes encoding pro-inflammatory and allograft rejection immune response pathways including upregulation of signaling pathways for TNFalpha and IL-2 were significantly increased in the DC1b *versus* DC1a cells as judged by normalized enrichment scores (NES)(**Supplementary Figure 1A**). Similar upregulation of genes encoding pro-inflammatory response pathways and cytokine signaling including interferon alpha and gamma, TNFalpha, or IL-2 were observed as judged by NES when DC2 cells were compared to DC1a cells or when DC2 cells were compared to DC1b cells (**Supplementary Figures 1B, C**). In contrast, expression of gene pathways encoding Myc targets and oxidative phosphorylation were reduced when comparing DC1b *versus* DC1a cells, and DC2 *versus* DC1a cells. Principal component analysis (**Supplementary Figure 1D**) also showed marked differences in gene expression patterns of the 3 DC populations with variances of 40% and 29% for PC1 and PC2 respectively. Duplicate experiments for purification of each of the 3 populations showed marked concordance of gene expression patterns within each population as compared to marked differences between populations (**Supplemental Figure 1E**).

Analysis of the upregulated genes shown in **Figures 4A-C** identified three surface proteins that were predicted to be upregulated in DC1b vs DC1a cells: CD324 (E-cadherin), DC1a vs DC2 cells CD207 (langerin), and DC1b vs DC2 cells (Ly6-D). Staining of the splenic DCs showed that the MFIs of the 3 receptors were increased the appropriate cell populations as predicted by the gene expression patterns (**Figures 4D–F**).

### All 3 DC Subsets Present Class I and II MHC Restricted OVA Peptide Antigens to OT-I CD8^+^ T Cells and OT-II CD4^+^ T Cells Respectively

We tested the ability of the 3 DC subsets to present a class I MHC restricted ovalbumin (OVA) peptide antigen to OT-1 CD8^+^T cells harvested from C57BL/6 mice bearing a transgenic TCR that recognizes this peptide antigen (41, 42). In addition, we tested the ability of the 3 DC subsets to present a class II MHC restricted peptide to OT-II CD4^+^ T cells with the appropriate transgenic TCR (42). Since the 3 DC subsets described above expressed both class I and class II MHC on their cell surfaces, we hypothesized that each DC subset would be able to present both the class I and class II restricted peptides to the OT-I and OT-II T cells respectively when the peptides were added to cultures of each DC subset with the appropriate T cells. However, the lower levels of class II MHC on DC1b cells as compared to the DC1a and DC2 cells, could reduce the activity of the former as compared to the latter subsets with regard to presentation of the class II restricted peptide.

**Figure 5:**
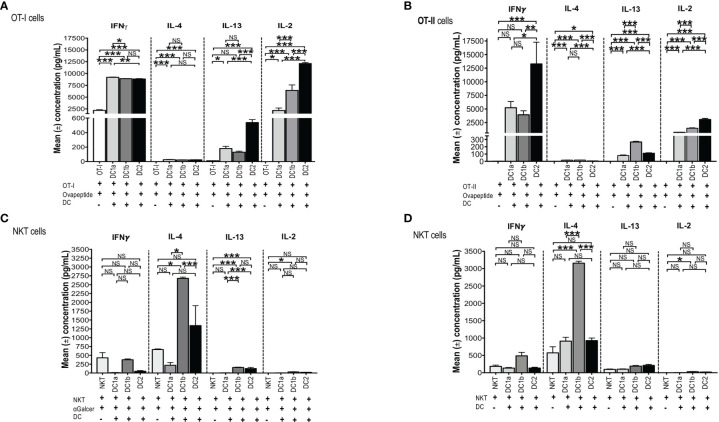
All 3 DC subsets can present Class I, and II MHC restricted OVA peptide antigens, but only DC1b cells present glycolipid antigens. **(A)** Mean concentrations of IL-2 (Right), IL-13 (Right Middle), IL-4 (Left Middle), and IFNγ (Left) in culture supernatants of FACS-sorted DC subsets (DC1a, DC1b and DC2) (1x10^4^ per well) from wild type untreated C57BL/6 mice that were cultured with OT-I restricted OVA_257-264_ peptide (1g/mL) and with CFSE-labelled OT-I CD8^+^ T-cells (1x10^5^ cells per well). In control experiments, CFSE-labelled OT-I CD8^+^ T-cells (1x10^5^ cells per well) were incubated with OVA_257-264_ peptide (1g/mL) but without DCs for 5 days. The concentrations of IL-4, IL-13, IL-2, IFN in culture supernatants was measured by Luminex. **(B)** Mean concentrations of IL-2 (Right), IL-13 (Right Middle), IL-4 (Left Middle), and IFNγ (Lef) in culture supernatants of FACS-sorted DC subsets (DC1a, DC1b and DC2) (1x10^4^ per well) from wild type untreated C57BL/6 mice that were cultured with OT-II restricted OVA_323-339_ peptide (10g/mL) with CFSE-labelled OT-II CD4^+^ T-cells (1x10^5^ cells per well). In control experiments, CFSE-labelled OT-II CD4^+^ T-cells (1x10^5^ cells per well) were incubated with OVA_323-339_ peptide (10g/mL) without DCs for 5 days. The concentrations of IL-4, IL-13, IL-2, IFN in culture supernatants was measured by Luminex. All data are representative of three independent experiments (6 mice per experiment). **(C, D)** Mean concentrations of cytokines in cultures of sorted DC1a (Pop 1), DC1b (Pop 2) and DC2 (Pop 3) (15x10^3^ cells/well) subsets incubated with sorted NKT cells (5x10^5^ cells/well) from untreated wild type BALB/c spleen cells. AlphaGalcer (α-galactosylceramide) (100ng/mL) was added to all cultures in **(C)** but not in **(D)**. Control cultures contained NKT cells with **(C)** or without **(D)** glycolipid but without DCs. Supernatants were harvested after 5 days, and concentrations were measured by Luminex. Data pooled from 2 or more independent experiments for a total of at least 10-12 mice per group, represented as mean ± SEM. *p < 0.05, **p < 0.01, ***p < 0.001, NS, not significant (p > 0.05). One-way ANOVA (Holm-Sidak’s multiple comparison test) was used for statistical analysis.

In order to test the antigen presenting activity of the 3 DC subsets independent of the need for antigen processing, OT-1 CD8^+^ T cells were harvested from the spleens of C57BL/6 transgenic mice and cultured in the presence or absence of sorted C57BL/6 DC1a, DC1b, or DC2 cells in the presence or absence of the class I MHC restricted SIINFEKL peptide derived from ovalbumin. Culture supernatants were assayed for IL-4, IFN, IL-13, and IL-2. **Figure 5A** shows that all 3 subsets of DCs stimulated the robust secretion of IFN and IL-2 with little or no secretion of IL-4 (<100pg/ml) and modest secretion of IL-13 (<600pg/ml). The mean concentrations of IFN (about 9,000 pg/mL) in the cultures containing the combination of CD8^+^T cells with each DC subset, and peptide were increased (p<0.001) as compared to the means in cultures containing the combination of CD8^+^ T cells and peptide without the DCs. DC1a cells stimulated a mean concentration that was a few hundred pg/mL higher than that of the DC1b and DC2 cells (p<0.05). In contrast, the DC2 subset stimulated the highest secretion of IL-2 (mean about 12,5000pg/mL) that was about 10,000 pg/mL higher than that stimulated by the DC1a subset (p<0.001).

In further experiments, we tested the ability of the 3 DC subsets to present a class II MHC restricted peptide to CD4^+^ OT-II T cells. **Figure 5B** shows that all 3 DC subsets stimulated robust secretion of IFN and IL-2 at levels that were comparable to those observed with OT-I cells (2,500-12,500pg/ml), and minimal secretion (<100pg/ml) of IL-4 or IL-13 (<300/pg/ml) consistent with the Th1 pattern observed with OT-I cells. The experiments with OT-II cells were repeated using ovalbumin protein instead of ovalbumin peptide. As shown in **Supplemental Figures 2A-D**, the Th1 pattern of OT-II cell immune response was similar when the protein was used instead of the peptide. In particular, the 3 DC subsets stimulated robust secretion of IFN gamma and IL-22 (>2,500pg/ml), and considerably less IL-2, IL-13, TNF alpha, and IL-17 (<1,000 pg/ml). The results indicate that all 3 DC subsets can process the protein for presentation to the OT-II T cells. Stimulation of the OT-I and OT-II cells by DCs pulsed with peptide or protein as measured by proliferation (CFSE dilution), indicated that all 3 subsets were effective inducers of proliferation (data not shown).

### The DC1b, but Neither the DC1a nor DC2 Subset, Presents Glycolipid to NKT Cells and Induces a TH2 Response

A previous study, showed that Batf-3 dependent CD8^+^ DCs were able to present a variety of glycolipids, including the potent activating Galcer glycolipid, to NKT cells *in vitro* and *in vivo*, but the Batf-3 independent DC2 cells could not (36). We hypothesized that the DC1b subset described above would be more potent than the DC1a and DC2 subsets in stimulating an immune response to the Galcer glycolipid after co-culture with purified NKT cells, since the DC1b subset expressed significantly higher levels of glycolipid antigen presenting molecule, CD1d, as compared to the DC1a and DC2 DC subsets (**Figure 2**).

To test this hypothesis, CD1dtetramer^+^ NKT cells were harvested from the spleens of BALB/c mice and cultured in the presence or absence of sorted BALB/c DC1a, DC1b, or DC2 cells in the presence of Galcer. **Figure 5C** compares the mean concentrations of IFN, IL-4, IL-13 and IL-2 from cultures of NKT cells and Galcer without or without the purified DC subsets. Only DC1b cells induced IL-4 (about 2,500 pg/mL) as compared to cultures lacking DCs. DC1b cells induced significantly higher levels of IL-4 as compared to DC1a and DC2 cells. DC2 cells failed to stimulate significantly increased production of IL-4 as compared to control cultures without DCs. **Figure 5C** shows that DC1b cells (but not DC1a) stimulated low levels of secretion of IL-13 (mean <100pg/ml) and IL-2 (mean <50pg/ml *in vitro* that were increased as compared to the background controls, but 25-50-fold less than IL-4.

Since, DCs are able to present constitutively expressed endogenous glycolipid antigens to NKT cells (48), the above experiments that tested the ability of DC subsets to stimulate cytokine secretion of purified NKT cells were repeated in the absence of Galcer. **Figure 5D** shows that the secretion of IL-4 induced by DC1b cells in the absence of Galcer was at least as robust as in the presence of Galcer. There was a highly significant increase in the mean concentration of IL-4 in NKT cell cultures with DC1b cells *versus* cultures with NKT cells alone (p<0.001). In addition, DC1a and DC2 cells failed to significantly increase IL-4 concentrations as compared to control cultures with NK T cells alone. A similar pattern was observed with IL-13 secretion; however, the concentrations were all below 200pg/ml. None of the 3 DC subsets induced robust NKT cell secretion of IFN or IL-2 (**Figures 5C, D**). In summary, only DC1b cells induced robust Th2 cytokine secretion by NKT cells that was predominantly polarized toward IL-4 in contrast to the TH1 pattern induced in OT-I and OT-II by all 3 DC subsets. The response of NKT cells induced by DC1b cells is likely due to endogenous glycolipid antigens.

### Slowing the Growth of B16-OVA Melanoma After Vaccination With OVA Loaded DC Subsets

**Figure 6:**
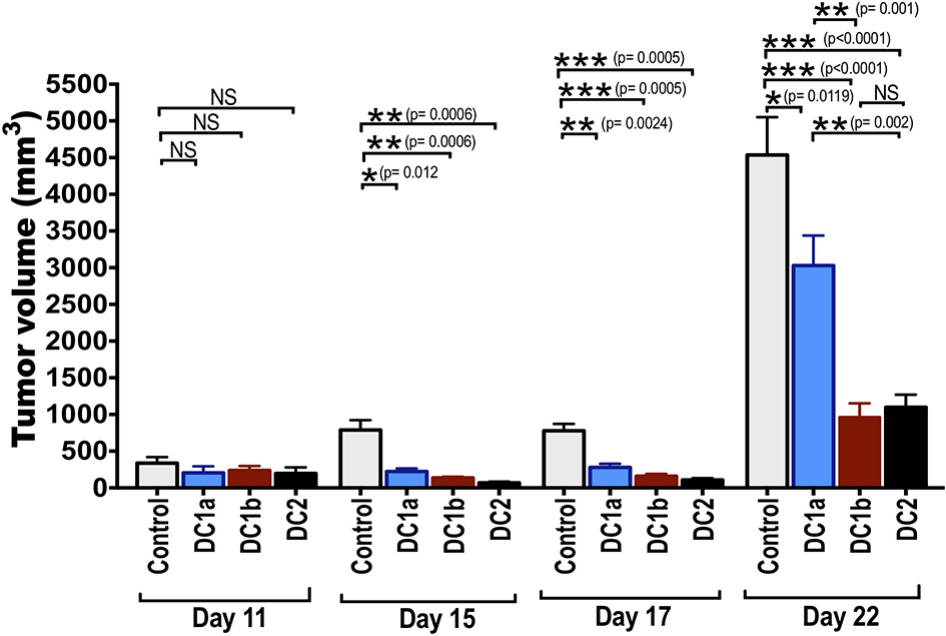
Tumor antigen-pulsed DC1b and DC2 cells are more effective than tumor antigen-pulsed DC1a cells at reducing tumor growth. Bar graphs depicting mean (+/-SE) tumor volumes in wild type C57BL/6 hosts monitored for 22 days. FACS-sorted DC subsets from wild type C57BL/6 mice were pulsed with whole OVA protein (500g/mL) *in vitro* for 1 hour, and then injected *in vivo* into wild type C57BL/6 hosts twice subcutaneously with a 2-week interval between injections. Hosts were then challenged with B16F10-ova tumor cells (5x10^4^ cells/mouse) injected subcutaneously on the flank of mouse 2 weeks after the second injection of OVA pulsed DCs, and the tumor volumes monitored over time. Control mice received tumor cells with no vaccination. Data are representative of two independent experiments. Each data point reflects data from 6-10 mice injected with FACS-sorted DC subsets pooled from 8-10 mice. One-way ANOVA (Holm-Sidak’s multiple comparison test) results are indicated as *P < 0.05, **p < 0.01, ***p < 0.001, NS, not significant (p > 0.05).

Batf3 dependent CD8^+^CD103^+^ DC1 cells, present in the microenvironment of tumors, cross-present tumor antigens to CD8^+^ T cells in the regional lymph nodes, and are required to initiate an immune response to the tumor (9–11). It is unclear, whether the DC1 cells are also required to initiate an anti-tumor immune response when the tumor antigens are incorporated into a subcutaneous vaccination to treat a tumor. In order to determine whether tumor antigen pulsed DC1a, DC1b, and/or DC2 cells can be used as a vaccination to initiate an immune response and slow the growth of the subcutaneous B16-OVA melanoma tumor, the purified subsets were incubated with ovalbumin whole protein and injected subcutaneously twice into wild type BALB/c mice 4 and 2 weeks before injection of the tumor cells. **Figure 6** shows the growth of the subcutaneous tumors as measured by tumor volume on days 11, 15, 17 and 22 after tumor cell injection. Control wild type mice had no vaccination.

The tumor growth in control mice was rapid, and the mean tumor volume at day 22 was about 4500mm^3^. Experimental mice that were vaccinated with the 3 different subsets of DCs showed significant slowing of tumor growth with all 3 subsets as compared to control mice. When DC1a cells were used as the vaccination, the day 22 mean volume was reduced to 2500 mm^3^ (p<0.05), and when DC1b and DC2 cells were used the volumes were further reduced to about 1,200 and 1,000 mm^3^ respectively (p<0.001). The DC1b and DC2 cells were significantly (p<0.01) more effective in slowing tumor growth than the DC1a cells.

## Discussion

Our findings show that Batf3 dependent CD8^+^DC1 cells, which had been previously distinguished from DC2 cells by the expression of surface markers such as CD24 and XCR1 (6, 20) and the nuclear transcription factor Irf8 (4, 5), can be further divided by their surface phenotype, gene expression profile and function into two subsets that we have designated DC1a and DC1b. Both DC1a and DC1b cells expressed CD24, XCR1, CD8, and Irf8 but the levels of expression were significantly different, and the DC1b cells expressed significantly lower levels. DC1b cells expressed significantly higher levels of CD1d, CD103, and Tim-4 as compared to the DC1a cells. In contrast, the DC2 cells failed to express CD24, XCR1, CD8, and Irf8, but did express Irf4. The DC1a, DC1b, and DC2 subsets were clearly identified by flow cytometry in the lymphoid tissues including the spleen, skin draining lymph nodes, msLNs, and PBMC when MHCII+CD11c+ total DCs were further analyzed for the expression of CD8 *versus* CD172.

Interestingly, only the DC1b subset was clearly identified by flow cytometry among total DCs in the intestines. The results suggest a failure of the DC1a and DC2 subsets to migrate to the gut. Our continuing studies investigate the chemokine receptors and other trafficking molecules on all three subsets, and whether sorted DC1b cells from the intestines differ in their gene expression pattern and function from the sorted DC1b cells from the lymphoid tissues. It has been previously shown that almost all CD8+DCs in the intestines express CD103 (27). The latter is consistent with the current finding that only DC1b cells are present in the intestines, since DC1b cells expressed significantly higher levels of CD103 than the DC1a or DC2 cells. E-Cadherin was also highly expressed along with CD103 on the DC1b cells, and this is likely to promote interactions between these cells since E-Cadherin binds to CD103, and the heterodimers can modulate their immune functions (48).

Sorted DC1a, DC1b and DC2 cells from the spleen were compared for their gene expression patterns using RNAseq analysis. Differences in gene expression patterns predicted that the expression of E-cadherin and Ly6-D surface receptors also could be used to distinguish the subsets, and this was confirmed by surface staining. Despite the capacity of all 3 DC subsets to present peptide and protein antigens to OT-II CD4 T cells and OT-I CD8 T cells, only the DC1b subset with the highest level of expression of the CD1d was able to effectively present the Galcer or endogenous glycolipid antigens to NKT cells. This finding is consistent with the previously reported ability of CD8^+^DCs to present a variety of glycolipid antigens to NKT cells *in vitro*, whereas the CD8^-^DCs were ineffective (36, 37). Interestingly, the NKT cell response elicited by the DC1b cells showed a Th2 skewing made up predominantly of IL-4 with minimal IFN or IL-2 (27). This polarization is likely due to the low level of CD40 on the DC1b subset, since blocking of the CD40/CD40L interaction has been shown to result in a Th2 polarization of NKT cells (49).

The DC2 cells were also more effective than DC1 cells in eliciting immune responses after vaccination with OVA pulsed DC subsets to control the growth of the OVA-B16 melanoma tumor cells. DC1 cells have been studied extensively for their capacity to cross-present intracellular tumor antigens and viral antigens to CD8^+^ T cells in local lymph nodes to subsequently induce systemic immune responses (9). However, the role of DC1 *versus* DC2 cells in the context of the induction of immune responses after subcutaneous vaccination with tumor or viral antigens remains to be elucidated, since the antigens in vaccines can be taken up directly by DCs in local lymph nodes without the need for cross-presentation. In addition, DCs can be pulsed *in vitro* with these antigens. Previous studies showed that DC2 cells can be at least as effective as DC1 cells in the induction of anti-tumor immune responses after vaccination, and can generate anti-tumor CD4^+^ and CD8^+^ memory T cells (35).

In the current study, we found that vaccination with OVA pulsed DC1b and DC2 cells were more effective than the pulsed DC1a cells in controlling the growth of the OVA-B16 melanoma. The results suggest that the DC1b cells are more effective in generating memory T cells to OVA tumor antigen than DC1a cells in the context of vaccination. However, further studies are needed to compare the efficacy of the two subsets for antigen cross-presentation of tumor antigens in the tumor microenvironment. Tumor control is likely to involve DCs in the generation of memory T cells from the vaccination as well as cross-presentation of antigen by DCs in the tumor microenvironment. In conclusion, our studies showed that CD8^+^Batf3 dependent DC1 cells are comprised of DC1a and DC1b subsets that differ in their surface marker phenotype, gene expression patterns, and function from each other as well as from DC2 cells.

## Materials and Methods

### Mice

Adult 8- to 10-week-old male *Batf3^-/-^* BALB/c (H-2K^d^)^1^ mice, wild type C57BL/6 (H-2K^b^), and (C57BL/6-Transgenic (*TcraTcrb*)1100Mj) OT-I (CD8^+^ TCR-Tag specific for ovalbumin (OVA) derived SIINFEKL peptide) mice (41) were used for the studies. Transgenic C57BL/6-OT-II mice (B6. Cg *(TcraTcrb*) 425Cbn/J) with a CD4^+^TCR-Tag specific for OVA _323-339_ peptide, were used also. All mice were obtained from the Jackson Laboratory (Bar Harbor, ME) (42). *Ja18^-/-^* BALB/c mice were originally obtained through a material transfer agreement with Dr. Taniguchi of RIKEN Research, Japan. Wild type BALB/c (H-2K^d^), *Cd1^-/-^* BALB/c mice and *Ja18^-/-^* BALB/c mice (39) were bred in the Department of Comparative Medicine, Stanford University (Stanford, CA), and all mice were maintained in the Department according to institutional guidelines approved by the National Institutes of Health.

### Immunofluorescent Staining and Fluorescent Activated Cell Sorter Analysis

Staining procedures and flow cytometry analysis performed on FACS LSRII or Aria machines (Becton Dickinson, San Jose, CA) and have previously been described in detail (43). The Fluorochrome conjugated mAbs used for staining were purchased from Invitrogen (San Diego, CA), Biolegend (San Diego, CA), BD biosciences (San Jose, CA), Novus Biologicals (Littleton, CA), Bio-Rad (Hercules, CA) and R&D Systems, (Minneapolis, MN). The following reagents and antibodies were used for flow cytometry:

CD11c (clone: N418; HL3); CD8a (clone: 53-6.7); CD205 (DEC205)(clone: NLDC-145); MHCII (I-A/I-E)(clone: M5/144.15.2); TLR3 (clone: 40C1285.6); TLR3 (clone: 40C1285.6); TLR4 (clone: MTs510); CD16 (FcγRIIIA)(clone: 5B11); CD32B (FcγRIIB); CD11b (clone: M1/70); CD172a (SIRPa) (clone: P84); CD45R/B220 (clone: RA3-6B2); CD370 (CLEC9A, DNGR1, clone: 7H11), CD103 (clone: 2E7), Treml4 (clone: 16E5), TIM-4 (clone: RMT4-54); CD40 (3/23); Rae-1; CD69 (clone: H1.2F3); TCRb (clone: H57-597); CD24 (clone: M1/69); XCR1 (clone: ZET); TIM-3 (CD366, clone: RMT3-32); CD1d (clone: 1B1); Clec9A (DNGR1, clone: 7H11); CD4 (clone: GK1.5); CD207 (clone: 929F3.01); CD324 (E-cadherin, clone: DECMA); Ly6-D (clone: 49-H4); PDL-2 (clone: TY25) and PDL-1 (clone: MIH5). Cells were stained with monoclonal antibodies and aqua dye (Zombie Aqua, Biolegend, San Diego, CA), a dead cell exclusion dye prior to flow cytometry analysis as per the manufacturer’s protocol (eBioscience, BD biosciences). Phycoerythrin and allophycocyanin conjugated CD1d-tetramers were obtained from the National Institutes of Health (NIH) Tetramer Facility, Rockville, MD. FACS analysis used FlowJo Software.

### Isolation, Purification, and Co-Culture of OT-I, and OT-II T Cells

The C57BL/6 spleen cells from either OT-I or OT-II transgenic mice (Jackson Laboratory) were harvested, and the single cell suspensions lysed with ACK lysis buffer (0.15M NH_4_Cl, 1mM KHCO3, and 0.1mM Na_2_EDTA {pH 7.4]) for 2 min at room temperature. Subsequently, cells were purified using conventional CD8^+^ T cell or CD4^+^ T cell isolation kit by negative selection on MACS LS columns, and then CFSE-labeled (labeled with 2.5μM CFSE) (Molecular Probe, Invitrogen, Eugene, OR). The DC subsets were co-cultured with enriched CFSE-labeled CD8^+^ T or CD4^+^ T cells from OT-I or OT-II mice spleen respectively with or without Ovalbumin peptides (OVA_257-264_ (1g/mL), OVA_323-339_ (10g/mL) or whole OVA protein (1g/mL) in 96-well round bottom plates at a ratio of 1:10 (DCs: T cells) at 37C, 5% CO_2_ in Complete (10% FCS) RPMI medium for 5 days. After co-culture the culture supernatants were collected for cytokine analysis using Luminex, and the cells were stained for OT-I or OT-II proliferation analysis by flow cytometry. OVA peptides and whole OVA protein were purchased from Sigma-Aldrich (St. Louis, MO).

### Isolation and Purification of NKT Cells

Invariant NKT (iNKT) cells were isolated using a gating strategy as described in detail before (44). Briefly splenocytes from wild-type BALB/c mice were stained with glycolipid loaded PE-conjugated CD1d tetramers (National Institutes of Health, Tetramer Facility, Emory-Atlanta, GA), and PE-conjugated-Galcer loaded CD1d dimer-mouse IgG fusion protein (BD Biosciences, San Jose, CA). iNKT cells were enriched subsequently by incubating with anti-PE microbeads (Miltenyi Biotech, Auburn, CA), and passing through a magnetic column (Miltenyi Biotech). The cells were then stained with anti-TCR-FITC and sorted by FACS Aria II (Becton Dickinson). The purity of sorted iNKT cells (TCR^+^CD1d-tet^+^) was more than 97%. Sorted iNKT cells were then cocultured *in vitro* with DC subsets from untreated wild type BALB/c mice.

### Isolation, Purification, and *In Vitro* Irradiation of DC Subsets

The C5BL/6 spleen cells from wild type untreated mice were cut into pieces with scalpel blades, and then digested with collagenase solution at 37C for 30 min on a shaker. The digested spleen cells were filtered with a cell strainer (70μm), and then RBCs were lysed with lysis buffer. For sorting, Miltenyi Mouse Pan-DC enrichment kit was used to enriched CD11c^+^ cells by magnetic beads labeling and purification on a LS column. For sorting DC cell subsets, the enriched CD11c^+^ cells were stained with the following monoclonal antibodies; CD11c, B220, MHCII, CD8a, and CD172a. The sorted DC subsets (DC1a (Pop 1), DC1b (Pop 2), DC2 (Pop 3) used for antigen (Ova) presentation to OT-I T cells, were irradiated (3,000 rads) from a ^137^Cs source (J.L. Shephard & Associates, San Fernando; CA).

### Cytokine Quantification of Supernatants From Cultures of NKT Cells, OT-I and OT-II Cells

To determine cytokine production in culture supernatants, NKT cells were cultured (at 5x10^4^ cells/well) with DC subsets (at 1x10^4^ cells/well), or alone. In another study of antigen presentation, OT-I cells (CD8^+^ T cells; 1x10^5^ cells/well), or OT-II cells or were cocultured with DC subsets (at 1.5x10^4^ cells/well) or alone for 4 days at 37 °C under 5% CO_2_ in 96-well flat-bottomed plates (Falcon, Becton Dickinson, Franklin Lakes, NJ) in a volume of 0.2 ml in Complete (10% FCS) RPMI medium for 4 days. Cell-free supernatants were collected after centrifugation for protein analysis, while cell pellets were resuspended and stained for flow cytometric analysis. Supernatants were assayed for IL-4, IL-13, IL-2 and IFN protein amounts using Multiplex magnetic Bead Array kit (ThermoFisher Scientific, Waltham, MA), quantified using a MAGPIX instrument (Luminex Corporation, TX).

### *In Vitro* Stimulation of NKT Cells by Splenic DC Subsets

To assess the efficacy of the 3 DC subsets to stimulate NKT cell cytokine production, sorted iNKT cells from untreated BALB/c mice, and sorted DC subsets *(*DC1a, DC1b, DC2) obtained from untreated wild type BALB/c spleen cells were cultured alone or together or in some cultures, α-galactosylceramide (100ng/mL) was added. iNKT cells (5x10^4^ cells/well) and DC subsets (1.5x10^4^ cells/well) were cultured at 37C, 5% CO_2_ in Complete (10% FCS) RPMI medium for 4 days. After culture, the supernatants were harvested, and the concentrations of cytokines in supernatants were quantified using a MAGPIX instrument (Luminex Corporation, TX).

### *In Vitro* Stimulation OT-I CD8^+^ T Cells, and OT-II CD4^+^ T Cells by Splenic DC Subsets

To evaluate antigen presentation capabilities of sorted DCs, irradiated DC subsets (DC1a, DC1b, and DC2) were plated in a round bottom 96-well plate at concentration of 1x10^4^ per/well. Purified OT-I CD8^+^ T cells, or OT-II^+^CD4^+^ T cells were mixed with DC subsets at a ratio of 1:10 and cocultured for 4 days at 37C in 5% CO_2_ in Complete (10% FCS) RPMI media. In some wells, MHCI restricted ovalbumin specific peptide SIINFEKL (OVA peptide _257-64_), or MHCII restricted ISQAVHAAHAEINEAGR (OVA peptide _323-339_, recognized by transgene encoded TCR of OT-II^+^CD4^+^ T cells), or whole OVA protein were added to the cell cultures. DC subsets were cultured either alone or with OVA or with OT-I T cells or OT-II cells. OT-I T, OT-II or were also either cultured alone or with OVA peptides or with whole OVA protein in control cultures.

### *In Vivo* Immunization of Mice With OVA Protein Pulsed DC Subsets Prior to Tumor (B16F10-OVA Cell Line) Challenge

The ability of DC subsets to process and present antigen to naïve T cells *in vivo* to control OVA-specific tumor (B16F10-Ova) was compared. Sorted DC subsets were pulsed *in vitro* with whole OVA protein (500g/mL) for 1hr in 37C, 5%CO_2_ incubator. Pulsed DCs were then used to vaccinate naïve wild type C57BL/6 hosts 2 times in a 2-week interval at a dose of 2x10^5^ cells per mouse by intravenous injection of the tail vein.

Two weeks after the last vaccination with primed DC subsets, C57BL/6 hosts were injected subcutaneously into the flank with 5x10^4^ B16F10-OVA cells/mouse and monitored for tumor growth. B16F10-OVA cells were kindly provided by Dr. Darrell Irvine (Massachusetts Institute of Technology MIT (Cambridge, MA). The equation used to calculate tumor volume was as follows: tumor volume = length x width^2^ x 0.52. Mice were euthanized when the tumor diameter of the tumor mass reached 2 cm.

### RNA Sequencing Analysis

Total RNA was prepared by sorting enriched CD11c^+^ subsets; DC1a (Pop 1), DC1b (Pop 2) and DC2 (Pop 3) from spleen cells (1,000 cells per sample) into RLT Plus lysis buffer (Qiagen) and stored at –80 °C, and then processed using RNeasy Micro Plus kit (Qiagen) per the manufacturer’s protocol. Two biological replicates were used for DC1a, DC1b and DC2 RNA-seq. The RNA quality was assessed by Agilent 2100 Bioanalyzer (Agilent Technologies). cDNA synthesis and amplification were prepared using SMARTseq v4 UltraLow Input RNA kit (Takara Inc.). The cDNA library was further processed for bulk RNASeq using a KAPA HyperPlus library kit (Roche Inc.) followed by ligation of Illumina Adaptor tags (Illumina Inc.). Library were quantified and sequenced at Human Immune monitoring Core Center and Genomics/Microarray Core Facility at Stanford University on Illumina Hiseq 4000. 

Sequencing yielded 76 bp paired-end reads with a mean sequencing depth of 24.8 million paired-end reads/sample. Trimmomatic (version 0.36) (45) was used to remove adaptors from reads. Clean reads were then aligned to the *Mus musculus* transcriptome (GRCm38) using histat2(version 2.1.0) (46) and gene count matrix of those mapped uniquely to known mRNAs (GRCm38.84.gtf) were generated by stringtie (version 1.3.1c, prepDE.py function) (47). DESeq2 was used to carry out differential gene expression of pairwise comparisons between tissues with the same genotype and between genotypes in the same tissue.

### Statistical Analysis

Results from independent experiments were pooled, and all data were analyzed using Prism (GraphPad Software; VERSION 7) by comparison of means using unpaired two-tailed Student’s *t* tests. A difference of 0.05 was accepted as statistically significant. The data in all figures represent mean ± SEM to indicate variation within each experiment. One-way ANOVA (Holm-Sidak’s multiple comparison test) was used for statistical analysis. *-p<0.05; **-p<0.01; ***-p<0.001; NS-not significant (p>0.05). Statistical analysis for RNA-seq data is described above.

## Data Availability Statement

The original contributions presented in the study are publicly available. This data can be found here: https://www.ncbi.nlm.nih.gov/geo/GSE181475.

## Ethics Statement

The animal study was reviewed and approved by Stanford University IACUC and APLAC committee according to institutional guidelines approved by the National Institutes of Health.

## Author Contributions

DH designed and performed research, contributed vital analytical methods, collected, analyzed and interpreted data, and wrote manuscript. PZ helped perform RNA sequencing and gene expression analysis. SD helped perform tumor experiment. RP helped with Luminex analysis. EM helped design experiments. EE helped design experiments, and helped write manuscript. SS provided overall research supervision and wrote manuscript. All authors contributed to the article and approved the submitted version.

## Funding

This work was supported by grants from the National Institutes of Allergy and Infectious Diseases (R01CA23395801), and National Heart, Lung, and Blood Institute (PO1HL- 075462).

## Conflict of Interest

The authors declare that the research was conducted in the absence of any commercial or financial relationships that could be construed as a potential conflict of interest.

## Publisher’s Note

All claims expressed in this article are solely those of the authors and do not necessarily represent those of their affiliated organizations, or those of the publisher, the editors and the reviewers. Any product that may be evaluated in this article, or claim that may be made by its manufacturer, is not guaranteed or endorsed by the publisher.

## Supplementary Material

Supplementary Figure 1 DC1a, DC1b and DC2 cells shows differences in expression of genes encoding immune response and cytokine signaling pathways. **(A–C)** GSEA (gene set enrichment analysis) of the most significantly (p < 0.1) upregulated and downregulated genes was compared using NES (normalized enrichment scores) of gene transcripts obtained by RNAseq analysis of sorted DC1a (Pop 1), DC1b (Pop 2) and DC2 (Pop 3) cells from WT BALB/c spleens. GSEA was used to determine NES as follows: - NES = actual ES/mean (ESs against all permutations of the data set). A small normal p value and a high FDR value indicates not significant. FDR is adjusted for gene set size and multiple hypothesis testing but not p value. **(D, E)**. Shows the PCA of the three DC subsets, and the distance among the samples respectively. The information for gene set analysis were obtained from the following websites. GSEA website: https://www.gseamsigdb.org/gsea/doc/GSEAUserGuideTEXT.htm#_Enrichment_Score_(ES) Hallmark gene sets were downloaded from website: http://bioinf.wehi.edu.au/MSigDB/v7.1/Mm.h.all.v7.1.entrez.rds: H hallmark gene sets.

Supplementary Figure 2 All 3 DC subsets pulsed with OVA protein stimulated robust secretion of Th1 cytokines by OT-II CD4^+^ T-cells. **(A–D)** Mean concentrations of IFN, IL-4, IL-13, IL-2, IL-22, TNF, and IL-17a in culture supernatants. FACS-sorted enriched DC subsets (DC1a, DC1b and DC2) (1x10^4^ per well) from wild type untreated C57Bl/6 mice cultured alone or with CFSE-labelled OT-II CD4^+^ T-cells from (1x10^5^ cells per well) in the presence of whole OVA protein (1g/mL). Cytokines in culture supernatants were measured by Luminex. Control cultures contained OT-II cells alone. Data was pooled from 2 or more independent experiments for a total of at least 10-12 mice per group. One-way ANOVA (Holm-Sidak’s multiple comparison test) results are indicated.

## Abbreviations

DCs, Dendritic cells; DC1, Type I dendritic cell; DC2, Type II dendritic cell; Batf3, basic leucine zipper transcription factor, ATF-3; NKT cell, Natural killer T cell; MLR, Mixed leukocyte reaction.
