# Supplementary figures and images for: Identification of Two Subsets of Murine DC1 Dendritic Cells That Differ by Surface Phenotype, Gene Expression, and Function
Source: Front Immunol. 2021 Oct 26;12:746469. doi: 10.3389/fimmu.2021.746469 (PMC8589020; doi:10.3389/fimmu.2021.746469)

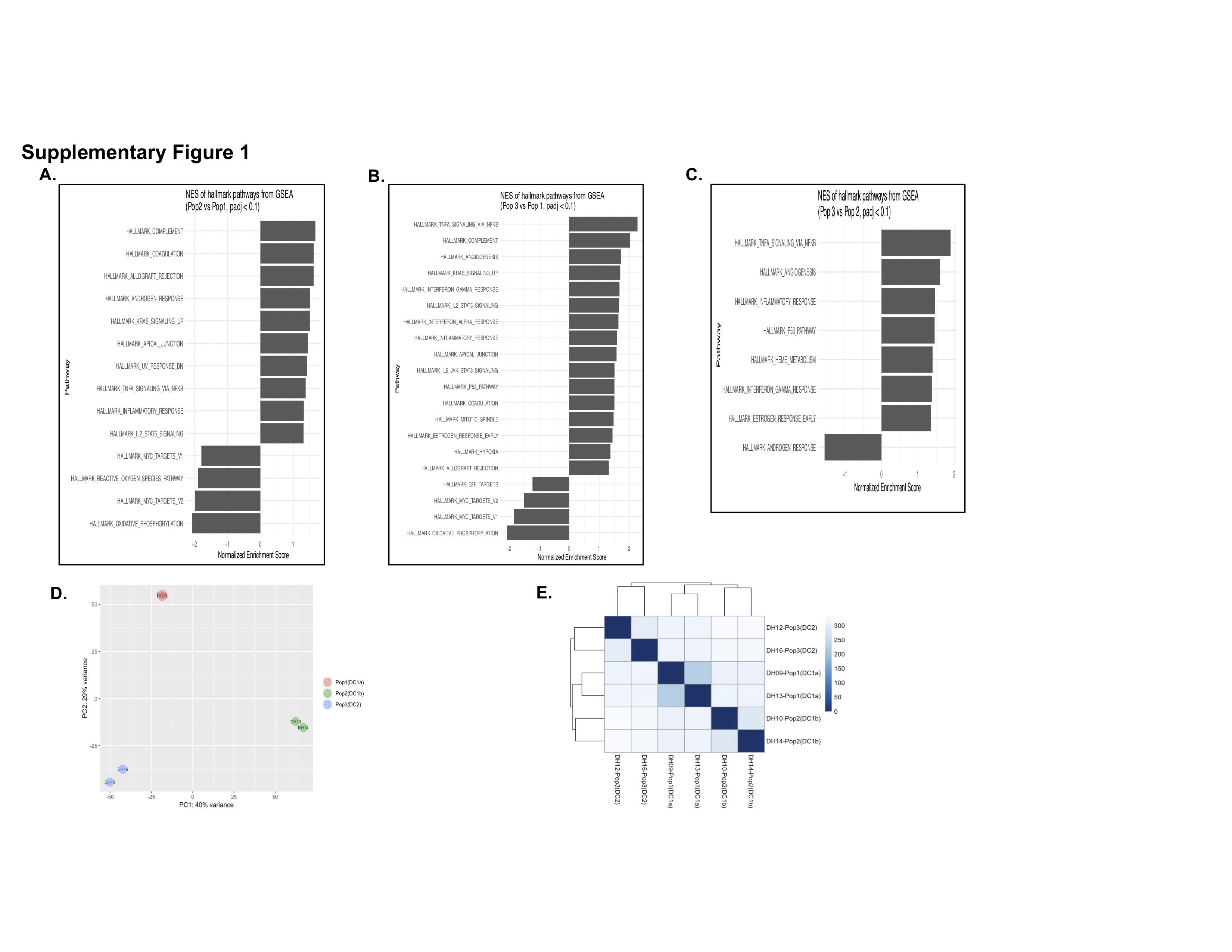

Supplement: Supplementary Figure 1 — DC1a, DC1b and DC2 cells shows differences in expression of genes encoding immune response and cytokine signaling pathways. (A–C) GSEA (gene set enrichment analysis) of the most significantly (p < 0.1) upregulated and downregulated genes was compared using NES (normalized enrichment scores) of gene transcripts obtained by RNAseq analysis of sorted DC1a (Pop 1), DC1b (Pop 2) and DC2 (Pop 3) cells from WT BALB/c spleens. GSEA was used to determine NES as follows: - NES = actual ES/mean (ESs against all permutations of the data set). A small normal p value and a high FDR value indicates not significant. FDR is adjusted for gene set size and multiple hypothesis testing but not p value. (D, E). Shows the PCA of the three DC subsets, and the distance among the samples respectively. The information for gene set analysis were obtained from the following websites. GSEA website: https://www.gseamsigdb.org/gsea/doc/GSEAUserGuideTEXT.htm#_Enrichment_Score_(ES) Hallmark gene sets were downloaded from website: http://bioinf.wehi.edu.au/MSigDB/v7.1/Mm.h.all.v7.1.entrez.rds: H hallmark gene sets. [file Image_1.jpeg]

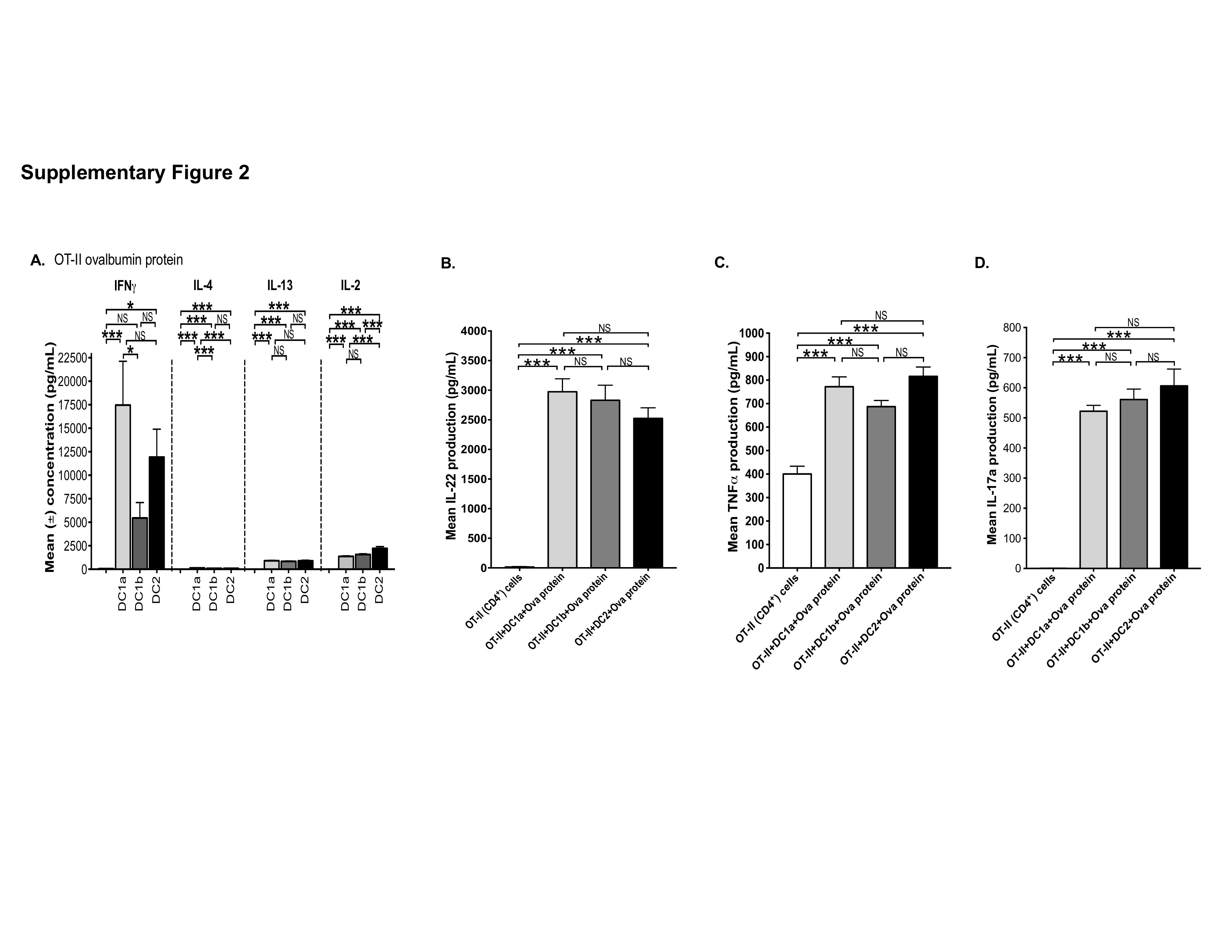

Supplement: Supplementary Figure 2 — All 3 DC subsets pulsed with OVA protein stimulated robust secretion of Th1 cytokines by OT-II CD4+ T-cells. (A–D) Mean concentrations of IFN, IL-4, IL-13, IL-2, IL-22, TNF, and IL-17a in culture supernatants. FACS-sorted enriched DC subsets (DC1a, DC1b and DC2) (1x10^4^ per well) from wild type untreated C57Bl/6 mice cultured alone or with CFSE-labelled OT-II CD4+ T-cells from (1x10^5^ cells per well) in the presence of whole OVA protein (1g/mL). Cytokines in culture supernatants were measured by Luminex. Control cultures contained OT-II cells alone. Data was pooled from 2 or more independent experiments for a total of at least 10-12 mice per group. One-way ANOVA (Holm-Sidak’s multiple comparison test) results are indicated. [file Image_2.jpeg]
